# Confronting Deep-Learning and Biodiversity Challenges for Automatic Video-Monitoring of Marine Ecosystems

**DOI:** 10.3390/s22020497

**Published:** 2022-01-10

**Authors:** Sébastien Villon, Corina Iovan, Morgan Mangeas, Laurent Vigliola

**Affiliations:** Institut de Recherche pour le Developpement (IRD), UMR ENTROPIE (IRD, University of New-Caledonia, University of La Reunion, CNRS, Ifremer), 101 Promenade Roger Laroque, 98848 Noumea, France; corina.iovan@ird.fr (C.I.); morgan.mangeas@ird.fr (M.M.); laurent.vigliola@ird.fr (L.V.)

**Keywords:** ecology, deep learning, artificial Intelligence, ecosystem monitoring

## Abstract

With the availability of low-cost and efficient digital cameras, ecologists can now survey the world’s biodiversity through image sensors, especially in the previously rather inaccessible marine realm. However, the data rapidly accumulates, and ecologists face a data processing bottleneck. While computer vision has long been used as a tool to speed up image processing, it is only since the breakthrough of deep learning (DL) algorithms that the revolution in the automatic assessment of biodiversity by video recording can be considered. However, current applications of DL models to biodiversity monitoring do not consider some universal rules of biodiversity, especially rules on the distribution of species abundance, species rarity and ecosystem openness. Yet, these rules imply three issues for deep learning applications: the imbalance of long-tail datasets biases the training of DL models; scarce data greatly lessens the performances of DL models for classes with few data. Finally, the open-world issue implies that objects that are absent from the training dataset are incorrectly classified in the application dataset. Promising solutions to these issues are discussed, including data augmentation, data generation, cross-entropy modification, few-shot learning and open set recognition. At a time when biodiversity faces the immense challenges of climate change and the Anthropocene defaunation, stronger collaboration between computer scientists and ecologists is urgently needed to unlock the automatic monitoring of biodiversity.

## 1. Introduction

In the age of climate change and anthropogenic defaunation [1,2] innovative methodology is needed to monitor ecosystems at large-spatial scales and high-temporal frequencies. Since the beginning of time, humans have learned from nature through visual observation, gradually using drawings, paintings, then photographs and videos. With the advent of low-cost digital cameras, modern ecologists can now visually gather visual data all around the planet, but they face a data processing bottleneck. While computer vision has long been used to speed up image processing, it is only since the emergence of deep learning (DL) algorithms that the revolution in the automatic assessment of biodiversity by video recording can be considered [3]. In fact, this revolution is underway, as shown by the exponential number of publications combining the words “biodiversity” and “deep learning” in a web of science bibliographic searches from 1975 to 2021, returning, on 28/10/2021, 175 publications, including zero before 2015, 12 from 2015 to 2017 and 118 since 2020.

The automation of video processing for biodiversity monitoring purposes is even more pressing in the oceans. Indeed, with 361 million km^2^, the oceans cover 71% of our planet and monitoring their biodiversity requires an immense effort only achievable through automation. Furthermore, due to notorious difficulties in observing underwater biodiversity (e.g., divers are limited by bottom time and by depth), video surveys have been increasingly used for decades in many habitats, with some examples in shallow reefs [4], sandy lagoons [5], deep seas [6] and in the pelagic ecosystem [7]. Global video surveys have even already been conducted. For instance, the FinPrint initiative had more than 15,000 video stations deployed in 58 countries in just three years for the first global assessment of the conservation status of reef sharks [8]. If the manual processing of such a large amount of videos was achievable for sharks at the cost of a massive labour effort, identifying and counting the abundance of thousands of other species in 15,000 video stations appears virtually impossible without automation. Unfortunately, the field of deep learning applied to marine biodiversity remains at a preliminary stage. A web of science bibliographic search combining the words “biodiversity”, “deep learning” and “marine” from 1975 to 2021 only returned 33 publications, including zero before 2016 and an average of 5.5 publications per year since then.

While DL algorithms have the potential to unclog the data processing bottleneck of video surveys, intrinsic characteristics of biodiversity, especially in the oceans, are in fact challenging this field of artificial intelligence, requiring special attention from computer scientists and strong collaboration with ecologists. Indeed, the current work of deep learning applications to automatically detect and classify animals in imagery is based on two premises: (1) an important database of each class of interest (thereafter “species”), and (2) a balanced dataset. These hypotheses are not verified for unconstrained wildlife video census. In particular, we point out three issues inherent to biodiversity video census, as well as the state of the art of DL approaches to answer them.

The aim of this work is (1) to point out ecological questions that can or cannot yet be tackled through DL applications by understanding its possibilities and limitations, and (2) to highlight recent advances of DL to unclog unconstrained wildlife video census.

## 2. Deep Learning for Biodiversity Monitoring

In recent years, a number of studies have examined the use of deep learning applied to ecological questions [9,10]. As shown in the most recent papers, the application of DL for species identification or detection relied on a limited number of species to process, as well as an important and balanced dataset. For instance, [11] discriminated 20 mammals species with an accuracy of 87.5% thanks to a dataset composed of 111,467 images; [12] detected and counted one species in videos with an accuracy of 93.4% with a dataset composed of 4020 images; [13] identified eight moth species with an F-measure (a common metric combining recall and precision) of 93% with a dataset of 1800 images, artificially augmented to 57,600 during the training of the model; [14] discriminated 109 plant species with an accuracy of 93.9% thanks to a dataset of 28,046 images. In the rare applications of deep learning on underwater videos [15], discriminated 20 coral reef fish species with an accuracy of 78% using a dataset of 69,169 images. Most proposals were adding information to DL models in order to reinforce the identification/detection, such as image enhancement, object tracking and class hierarchy. Historically, DL models were trained on benchmarks, composed of hundreds to thousands of images per class, and applied to a closed testing dataset [16] A closed dataset is defined as classes in the testing dataset that are the same as classes in the training dataset. Thus, deep learning models trained for biodiversity monitoring still rely on closed, relatively large and balanced collection of images, following the framework developed in the field of computer science without considering intrinsic properties of biodiversity.

## 3. Biodiversity Rules and Deep Learning Limits

Species are no simple objects to classify. Their distribution and abundance follow a few universal rules that need to be accounted for in order to unlock the automatic assessment of biodiversity in underwater videos. 

Since the early work of [17,18,19], ample evidence across world ecosystems show that in nearly every community in which species have been counted, the distribution of species abundance is highly skewed, such that a few species are very abundant, and many species are present in relatively low numbers. For deep learning applications, this first universal rule of ecology implies heavily unbalanced training datasets, while balanced datasets are a crucial part of robust and accurate models. This issue, hereafter referenced as the “long-tail dataset issue”, is especially acute for speciose communities, such as coral reef fishes, where several hundred species, of which only a few dozen are abundant, can co-occur at a single site and at a single time in a video or another sampling station [20,21].Intimately linked to the first rule, a second universal rule of ecology was proposed by [22] based on the early work of [23,24]. It states that species abundance is highest near the centre of their geographic range or environmental niche and then declines towards the boundaries. Thus, species tend to be scarce near the limits of their distribution. More generally, rarity is an intrinsic characteristic of biodiversity, with most communities composed of a large number of rare species. For deep learning, species rarity implies a lack of training images for a large part of species, where only a few or one individual can be seen in hundreds of hours of videos. This issue, hereafter referenced as the “scarce data issue”, is significantly marked in species-rich assemblages, such as coral reef fishes, where most species are demographically rare [20,21]. 

The third rule of ecology that seems relevant for deep learning stems from the openness of ecosystems [25,26]. The flow of energy, material, individuals and species across ecosystem boundaries is ubiquitous and plays a key role in ecosystem functioning. The degree of openness of marine ecosystems is particularly high because of the aquatic environment that facilitates the movement of species and the existence of most marine species of a planktonic larval stage favouring dispersal [27].Ecosystem openness implies the issue of applying a deep learning model to an “open world problem”. According to [28],this issue is intrinsic to DL and is defined by a greater number of classes (in our case, species or conditions) in the application dataset than in the training dataset. In the context of biodiversity monitoring, the application dataset is composed of unconstrained recordings of wildlife and ecosystems, and due to ecosystem openness and the limits of sampling and annotation efforts, it cannot be considered as a closed-world application.

**Figure 1:**
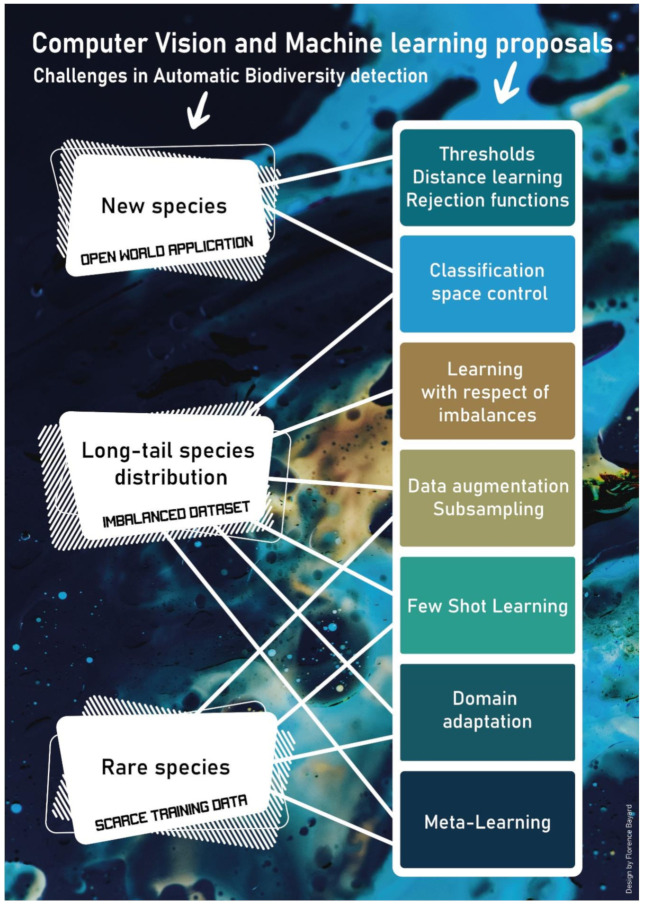
Ecological rules, their impacts on machine learning and state-of-the-art proposal to answer it.

By comparing the current state-of-the-art of deep learning applications for biodiversity monitoring with some universal rules of biodiversity, we highlighted three problems inherent in such methods that remain to be solved in order to unlock the automatic assessment of biodiversity in underwater videos. We now discuss some potential solutions to these issues (Figure 1).

## 4. Long-Tail Datasets

Long-tail datasets are problematic for deep learning model training. Classes with more samples in the training dataset have more impact on the final model. As a result, a model trained with an imbalanced dataset will have more success in predicting classes with more data, while predicting classes with fewer data will be hampered [29,30,31]. Furthermore, it was shown [32] that the degradation of predictions due to imbalanced datasets increases as the complexity of the task increases, which makes data imbalance of great impact in the case of complex ecosystems studies. Although a few studies suggested that training quality (e.g., sufficient data of all classes in datasets) can decrease the impact of imbalances [31,33], it is impossible to gather enough data for the large number of “rare” species composing marine ecosystems.

Two major ways of tackling this issue have emerged in the literature.

The first approach to address the long-tail dataset issue consists in balancing the dataset itself, for which the most popular methods are data augmentation or data generation. The technique of subsampling, which removes data from classes with large samples is not considered here because it wastes a large amount of useful information.

Data augmentation consists of artificially augmenting the number of images of classes with fewer data in order to increase their impact on the model training [34]. There are numerous methods to increase images datasets, such as resampling, geometric transformations, kernel filters (sharpness, colours and blurring) or feature space augmentation [35,36]. Apart from resampling, which simply uses the same image multiple times during the training phase, data augmentation consists in transforming existing images in the dataset and induces changes in order to mimic changing conditions and limit the overfit of DL models.

Data generation, through a generative adversarial network (GAN), variational autoencoder (VAE) or neural style transfer, is another way to increase a dataset’s size [35,37,38,39,40,41]. GANs are Deep Neural Networks (DNN), which, through learning thanks to existing data, are able to generate new images in the same representation space (i.e., images that “look like” those of the GAN training dataset). However, similarly to data augmentation, it is important to induce variations in the artificial dataset to prevent overfitting. Data can also be generated through visual engines, such as Unreal or Unity.

In the field of object detection, a few studies have directly cropped out objects of their original scenes and pasted them in new scenes [42,43]. Furthermore, a number of studies have simultaneously used both data augmentation and data generation [44]. 

The second approach to tackle the long-tail dataset issue consists in accounting for the imbalance of samples for each class in the training algorithms itself [45]. The focal loss, introduced by [46], adds to the Cross-Entropy (CE, a value used to improve deep model predictions during the training stage) two variables taking into account the ability of the networks to discriminate all classes and the proportion of each class in the dataset. In the same spirit, [47,48,49] propose to modify the CE with respect to dataset imbalances. Furthermore, [48] propose to control the classification space and margin between classes to boost the classification of classes with few data.

It has been shown that such methods can significantly improve the accuracy of DL model predictions [41].

## 5. Scarce Data

Deep learning models are efficient when a reasonable number of images per class is available during the training phase. However, ecosystems are composed of a large part of rare species, with only a few images in training datasets (e.g., the tail of species distribution).

The current popular proposal to unlock DL training with limited datasets is Few-Shot Learning (FSL), which builds algorithms able to discriminate classes with very few samples, classically from 1 to 20 images per class. Finn et al. [50] was a precursor of FSL and was based on a phase of meta-training, during which the model was trained on a different task at each iteration. To build a model able to discriminate five classes, at each training iteration, five classes were randomly selected from among the 64 possible training classes. This phase enabled the model to “learn to learn” and to adapt itself to new tasks. Once the meta-training task was completed, the model could be adapted to a new task with very few images. More recently, [51,52] proposed to improve meta-learning by tweaking the meta-training or training batch compositions. 

Apart from meta-training, matching network and metrics learning are other popular options [53,54,55,56].Such methods aim to train a model to match a “Support Set” (i.e., a small training dataset composed of 1–5 images per class) with a “Query image” (i.e., the image to predict the label for). These approaches are two-fold: (1) they require a DL model able to manage the classification space with few images, and (2) they require the training of a robust metric to measure distances between the Query Image and the different Support Set clusters. More details on FSL can be found in the more exhaustive [57] review.

FSL is usually limited to 5–20 classes. However, recent papers are trying to overcome this limitation with approaches known as Many Classes Few-Shots [58,59] by leveraging a possible hierarchy among classes. This could be an opportunity for ecological applications, as there is a known hierarchy between species (i.e., taxonomy). Unfortunately, to date, most FSL algorithms are not able to discriminate more than 20 classes or with low accuracy [60]. Moreover, the study of methods for both imbalanced datasets and scarce data is for now limited to traditional benchmarks (such as imageNet [61] or mnist (http://yann.lecun.com/exdb/mnist/)(accessed on 1 June 2021).) without ecological questions. However, FSL’s application to underwater videos was first trialled in 2021 [62].

## 6. Open World Application

Applying a DL model of animal detection and/or species identification in the wild necessarily implies an “Open World” application. Indeed, DL algorithms, by nature, optimise a global function capable of discriminating several known classes of interest [16]. For species detection and identification, the algorithms also have to discriminate such classes from the background [63]. Unfortunately, it is not possible to predict the behaviour of a DL model when facing objects unseen during the training phase (e.g., new species, new morphologies, weather conditions, seascapes). 

Approaches to tackling open world issues are regrouped under the Open Set Recognition (OSR) proposal, whether applied to Machine Learning [64,65,66,67,68,69,70,71,72,73] or Deep [67,69,74,75,76,77,78,79,80,81]. OSR is a growing field of research that has only been studied since the last decade. 

Proposals on OSR rely on three principles that can be associated. First, managing the classification space in order to maximise inter-class margin and minimiz\se intra-class spaces occupation. Such managing will create dense clusters of classes representation. Second, distances are chosen or learned through machine learning to evaluate new images with respect to learned clusters. Third, thresholds are selected or learned through machine learning to discriminate “known classes” from “new classes” with respect to the chosen distance. The overarching assumption is that images of new classes will be classified in the unused classification space, away from learned classes’ clusters. Applied to DL, most efficient methods rely on “OpenMax”, proposed by [74] and extended by [75,76], which replaces the usual classification layer of deep architecture known as “SoftMax”. The SoftMax function transforms the activation vector (i.e., the last feature vector of a Deep Network input) into a vector of *n* values, with *n* being the number of classes to discriminate. OpenMax adds a rejection function to SoftMax. This rejection function relies on the distance computed between previously learned data and the new input. The two main risks of OSR approaches are (1) depending on a training dataset to learn something that is not present in the training dataset and (2) potential overfit through the minimisation of potential classification space for learned classes. We also noted that most approaches are applied to image classification issues, and very few works cover object detection. 

To date, there is only one research article [82] trying to resolve the three issues of imbalanced datasets, scarce data and the open world at the same time for a classification problem. Yet, in order to be robust to real life applications, DL needs to move from its initial challenge of discriminating a limited number of classes with balanced and numerous data to a more realistic imbalanced, scarce and open data distribution.

## 7. Conclusions

Deep learning applications for biodiversity monitoring have been increasingly explored since 2017 [9] and are still in their early stages in marine realm applications. However, most studies rely on methods designed for and tested on generic benchmarks, which restrains the field of applications. To become an efficient tool for unconstrained wildlife census and conservation monitoring, collaborative research in computing science and ecology shall account for some of the universal rules of biodiversity. In this perspective, we highlighted methods from the state-of-the-art of artificial intelligence. Such methods have the potential to overcome the current limits of automatic video processing by focusing more thoroughly on the topics of imbalanced datasets, scarce data and open-world application. As such, efficient deep networks working with few data, such as few-shot learners and one-shot learners, improvement of the robustness to data imbalances through a specifically built learning process, and the ability to treat information absent from the training datasets with Open Set Recognition paves the way for an interdisciplinary branch of science between computer sciences and ecology. Rather than merely transferring the DL methods originally developed to perform on benchmarks to ecological questions, ecologists and computer scientists should foster their collaborations at the interface of both disciplines. As such, DL algorithms would become question-driven instead of adapted, which could leverage the immense challenges that biodiversity is facing with climate change and the Anthropocene defaunation. Conversely, ecologists may have a great interest in understanding the full potential offered by artificial intelligence techniques in order to develop new indicators that require too many human resources to operate until now or for lack of available data.
